# Heat shock transcriptional factors in *Malus domestica*: identification, classification and expression analysis

**DOI:** 10.1186/1471-2164-13-639

**Published:** 2012-11-20

**Authors:** Filomena Giorno, Gea Guerriero, Sanja Baric, Celestina Mariani

**Affiliations:** 1 Research Centre for Agriculture and Forestry Laimburg, Laimburg 6, Auer/Ora, BZ, 39040, Italy; 2 Department of Molecular Plant Physiology, Radboud University Nijmegen, Heyendaalseweg 135, Nijmegen, 6525 AJ, The Netherlands

**Keywords:** Hsf, *Malus domestica*, Gene expression, High temperature, Apple fruit/ flower

## Abstract

**Background:**

Heat shock transcriptional factors (Hsfs) play a crucial role in plant responses to biotic and abiotic stress conditions and in plant growth and development. Apple (*Malus domestica* Borkh) is an economically important fruit tree whose genome has been fully sequenced. So far, no detailed characterization of the Hsf gene family is available for this crop plant.

**Results:**

A genome-wide analysis was carried out in *Malus domestica* to identify heat shock transcriptional factor (*Hsf*) genes, named *MdHsfs*. Twenty five *MdHsfs* were identified and classified in three main groups (class A, B and C) according to the structural characteristics and to the phylogenetic comparison with *Arabidopsis thaliana* and *Populus trichocarpa.* Chromosomal duplications were analyzed and segmental duplications were shown to have occurred more frequently in the expansion of *Hsf* genes in the apple genome. Furthermore, *MdHsfs* transcripts were detected in several apple organs, and expression changes were observed by quantitative real-time PCR (qRT-PCR) analysis in developing flowers and fruits as well as in leaves, harvested from trees grown in the field and exposed to the naturally increased temperatures.

**Conclusions:**

The apple genome comprises 25 full length Hsf genes. The data obtained from this investigation contribute to a better understanding of the complexity of the Hsf gene family in apple, and provide the basis for further studies to dissect Hsf function during development as well as in response to environmental stimuli.

## Background

Trees are sessile organisms with long lifespans that regularly experience climatic fluctuations in their native environment. Therefore, survival and reproduction is dependent upon an array of protective mechanisms that involve the activation of a wide range of transcriptional factors, and their products are considered to play a central role in response to extreme physiological conditions. There is evidence that members of the heat shock transcriptional factor (Hsf) family are important regulators in sensing and signaling of different environmental stresses [1]. Similarly to many other transcription factors, the Hsfs have a modular structure containing signature domains structurally and functionally conserved throughout the eukaryotic kingdom. A common core structure in the Hsfs is composed of an N-terminal DNA binding domain (DBD), characterized by a central helix-turn-helix motif that specifically binds to the heat shock elements (HSE) in the target promoters, and an adjacent bipartite oligomerization domain (HR-A/B) composed of hydrophobic heptad repeats [2]. Hsf trimerization via the formation of a triple stranded alpha-helical coiled-coil is a prerequisite for high affinity DNA binding and, subsequently, for transcriptional activity. Other Hsf functional modules include clusters of basic amino acids essential for nuclear import (NLS), leucine-rich export sequences important for nuclear export (NES), and a less conserved C-terminal activator domain (CTAD) rich in aromatic, hydrophobic and acidic amino acids, the so-called AHA motifs [2,3].

In contrast to *Saccharomyces cerevisiae*, *Caenorhabditis elegans*, and *Drosophila melanogaster*, that each possesses only a single Hsf gene, plant genomes contain large numbers of *Hsf* genes, up to 52 [1,4,5]. Based on structural characteristics and phylogenetic comparisons, plant Hsfs are grouped into classes A, B and C [2,6]. All class A and C Hsfs have an extended HR-A/B region due to the insertion of 21 (Class A) or seven (class C) amino acid residues between A and B parts of the HR-A/B region. On the contrary, in class B Hsfs, the HR-A/B region does not contain insertions. In addition, sequence comparisons and structural analyses indicate that the combination of a AHA motif with an adjacent nuclear export signal NES represents a peculiar signature domain for many plant class A Hsfs [6,7].

After the release of the whole genomic sequences of several plant organisms, including rice (*Oryza sativa*), maize (*Zea mays*), poplar (*Populus trichocarpa*), medicago (*Medicago truncatula)*, tomato (*Solanum lycopersicon)*, the Hsfs family was analyzed extensively, both to place each member in an organized nomenclature system and to provide maps of their expression [7-10].

Recently, the full genome sequence of the domesticated apple *(Malus domestica* Borkh) has been published [11]. This provides a useful genomic tool to study this economically important fruit crop. As transcriptional factors, Hsfs are involved in different aspects of plant life including tolerance to biotic/abiotic stresses and developmental processes [12-14]. Therefore, this gene family represents an important group of transcriptional factors to investigate and to characterize. Genome scale analyses of the transcriptional response during development and to environmental stimuli require a precise and complete annotation of genes in order to provide reliable and exhaustive data. Therefore, the aim of this study was to annotate the full length *Hsf* genes in apple, and to analyze their expression profiles by quantitative real time PCR (qRT-PCR) in different organs/tissues from plants grown in the field and exposed to natural environmental conditions. The results of this work provide a foundation to better understand the functional structure and genomic organization of the *Hsf* gene family in apple, and will be undoubtedly useful in future gene cloning and functional studies.

## Results

### Identification, classification and duplication of *Hsf* genes in the *Malus domestica* genome

CDS sequences corresponding to putative *Hsf* genes from *Malus domestica* (*MdHsfs*) were searched in the Apple Genome v1.0 [15]. As a result, 36 genes encoding for putative MdHsfs proteins were identified. All candidate MdHsf proteins were surveyed, and incomplete sequences for the DBD domain and for the remaining functional domains were removed. This resulted in the selection of twenty five complete sequences. These *MdHsf* genes were distributed on 12 of the 17 apple chromosomes with the largest number, comprised of six *Hsf* genes, detected on chromosome 15 (Table 1). According to the multiple sequence alignment of the DBD and HR-A/B region, 16 genes were determined to be Class A, seven genes were identified as Class B and two were classified as Class C.

**Table 1:** **List of *Hsfs *genes in the *Malus domestica *genome**

**Gene name**	**Chromosomal localization**		**Size** (**aa**)	**MW**(**kDa**)	**pI**
*MdHsfA1a*	*Chr6*	MDP0000517644	540	59.37	4.76
*MdHsfA1b*	*Chr10*	MDP0000156337	546	61.14	4.96
*MdHsfA1c*	*Chr13*	MDP0000232623	550	60.07	6.01
*MdHsfA1d*	*Chr16*	MDP0000259645	580	64.34	5.04
*MdHsfA2a*	*Chr8*	MDP0000489886	380	42.42	4.73
*MdHsfA2b*	*Chr15*	MDP0000243895	377	42.24	4.63
*MdHsfA3a*	*Chr12*	MDP0000131346	516	56.26	4.18
*MdHsfA3b*	*Chr14*	MDP0000606400	455	50.37	6.43
*MdHsfA3c*	*Chr14*	MDP0000174161	582	64.34	4.89
*MdHsfA4a*	*Chr5*	MDP0000155849	420	47.23	5.62
*MdHsfA5a*	*Chr9*	MDP0000301101	483	53.81	5.08
*MdHsfA5b*	*Chr15*	MDP0000613011	482	54.19	5.48
*MdHsfA8a*	*Chr10*	MDP0000191541	414	46.89	4.55
*MdHsfA8b*	*Chr13*	MDP0000172376	411	44.86	5.10
*MdHsfA9a*	*Chr2*	MDP0000194672	713	75.89	6.86
*MdHsfA9b*	*Chr15*	MDP0000319456	482	53.29	4.86
*MdHsfB1a*	*Chr2*	MDP0000527802	294	32.26	8.76
*MdHsfB1b*	*Chr15*	MDP0000578396	232	28.40	4.67
*MdHsfB2a*	*Chr1*	MDP0000155667	276	30.97	5.96
*MdHsfB3a*	*Chr12*	MDP0000622590	243	27.77	7.22
*MdHsfB3b*	*Chr14*	MDP0000202716	243	27.82	7.82
*MdHsfB4a*	*Chr8*	MDP0000209135	381	42.85	7.62
*MdHsfB4b*	*Chr15*	MDP0000129357	383	43.19	7.64
*MdHsfC1a*	*Chr2*	MDP0000230456	324	36.25	6.27
*MdHsfC1b*	*Chr15*	MDP0000320827	344	38.36	5.02

MW: molecular weight; pI: Isoelectric point.

**Figure 1:**
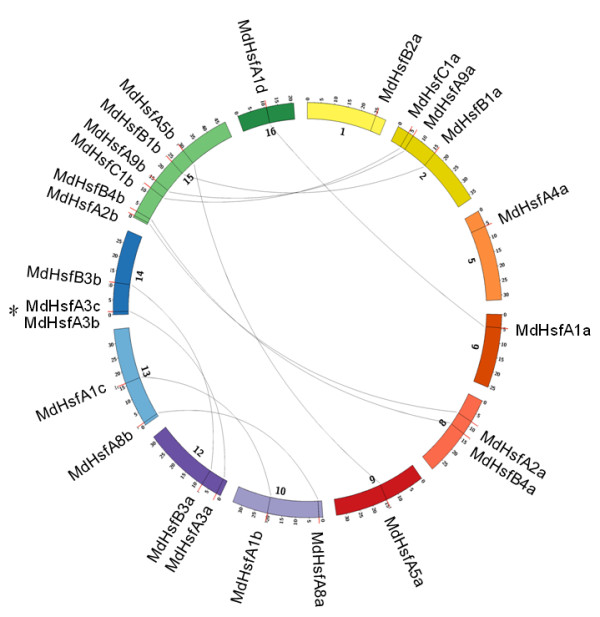
**Localization and duplication of the *Hsf *genes in the apple genome.** Circular visualization of the 25 *Hsfs* mapped on the different chromosomes in the apple genome was obtained using the Circos software. Picture shows only the chromosomes containing *MdHsf* genes, and chromosome number is indicated on the inner side. Segmental duplications were joined by the lines, while the tandem duplication of *MdHsfA3b* and *MdHsfA3c* is indicated by an asterisk.

Gene duplication events have been indicated as an important mechanism in the evolution of plant genomes [16]. Therefore, duplications of *MdHsfs* were also analyzed. As shown in Figure 1, a total of 12 duplicated gene pairs of *MdHsfs* were identified, including 11 segmental duplication events between chromosomes (*e.g. MdHsfC1a* and *MdHsfC1b*) as well as one tandem duplication event within the same chromosome, *e.g. MdHsfA3c* and *MdHsfA3b*. *MdHsfA3c* was the only *Hsf* involved in both duplication events, as it was duplicated with *MdHsfA3b* in tandem on chromosome 14 and also segmentally duplicated with *MdHsfA3a* on chromosome 12.

### Analysis of conserved domains in the apple Hsf proteins

Prediction of the typical signature domains present in the MdHsfs protein sequences was carried out by comparing the identified apple Hsfs to those of homologous, well characterized proteins of model plants such as tomato or Arabidopsis [2,6,7]. Table 2 lists five conserved motifs that were identified by sequence alignment, and their positions in the protein sequences. All the MdHsfs showed the presence of the highly conserved DBD domain in the N-terminal region, consisting of a three-helical bundle (H1, H2 and H3) and a four-stranded antiparallel β-sheet. The length of the DBD motif was quite variable with the smaller size observed for MdHsfB1b. The presence of the coiled-coil structure characteristic of leucine-zipper type protein interaction domains, which is a property of the HR-A/B region, was instead predicted in all MdHsfs proteins by using MARCOIL tool. Furthermore, the majority of the MdHsfs showed the presence of NES and NLS domains which were described to be essential for shuttling Hsfs between nucleus and cytoplasm [7]. Additional sequence comparison allowed the identification of AHA motifs in the center of the C-terminal activation domains, as it is expected in the A-type Hsfs. By contrast, these domains were not identified in the B and C-type MdHsfs.

**Table 2:** Functional motifs of apple Hsfs

**Gene name**	**DBD**	**HR**-**A**/**B**	**NLS**	**NES**	**AHA**
*MdHsfA1a*	39-132	154-220	(242) NKKRRLKK	(502) MDNLTEKMG	AHA (454) DIEAFLKDWDD
*MdHsfA1b*	17-152	174-240	(264) NKKRRLPR	(529) MNHITEQM	AHA (482) DIFWEQFLTAS
*MdHsfA1c*	16-156	173-243	(268) NKKRRLPR	(533) MNHITEQMQ	AHA (486) DIFWEQFLTAS
*MdHsfA1d*	103-196	217-284	(307) NKKRRLKR	(563) MDNLTEKMG	AHA (516) DIEAFLKDWDD
*MdHsfA2a*	30-132	147-213	(228) KNRK-X_7_-RKRR	(368) LLDQMGYQ	AHA1 (318) ETIWEELWSD AHA2 (360) DWGKDLQD
*MdHsfA2b*	38-131	145-212	(227) KNR-X_6_-RKRR	(365) LVDQMGYL	AHA1 (315) ETIWEELWSD AHA2 (355) DWGEDLQD
*MdHsfA3a*	99-209	226-285	(297) KTRRKFVK	nd	AHA1 (431) EDIWSMGFGV AHA2 (450) ELWGNPVNY AHA3(470) LDVWDIGPLQ AHA4 (486) IDKWPAHDS
*MdHsfA3b*	99-232	253-312	(328) KDIGSSRVRRKFVK	nd	nd
*MdHsfA3c*	99-244	265-324	(340) KDIGSSRVRRKFVK	nd	AHA1 (500) EDIWSMNFDV AHA2 (518) NELWGNPXNY AHA3 (539) LDVWDIDPLQ AHA4 (555) INKWPAHES
*MdHsfA4a*	10-103	123-190	(208) RKRRLPR	(407) LTEQMGHL	AHA1 (252) LTFWEDTIHD AHA2 (356) DGFWEQFLTE
*MdHsfA5a*	12-105	116-183	(194) RK-X_10_-KKRR	(477) AETLTL	AHA (431) DVFWEQFLTE
*MdHsfA5b*	12-105	117-183	(194) RK-X_10_-KKRR	(477) AETLTL	AHA (431) DVFWEQFLTE
*MdHsfA8a*	18-111	129-199	(177) RNRLR	(389) TEQMGHL	AHA (308) DGAWEQFLLA
*MdHsfA8b*	18-111	127-196	(172) RLLRNR	nd	AHA (306) DGAWEQLLLG
*MdHsfA9b*	139-239	241-308	(324) KR-X_8_-KRRR	(258) LKADQD	nd
*MdHsfA9b*	139-239	241-308	(324) KR-X_8_-KRRR	(258) LKADQD	nd
*MdHsfB1a*	6-99	142-191	(246) KGDEKMKGKK	nd	nd
*MdHsfB1b*	2-35	78-127	(181) KGEEKMKGKK	(159) LDMEGG	nd
*MdHsfB2a*	22-115	154-197	(167) RLRK	nd	nd
*MdHsfB3a*	19-112	149-194	(223) RKRKR	(208) PKLFGVRLE	nd
*MdHsfB3b*	22-116	149-194	(179) KRKCK (223) RKRKR	(208) LKLFGVRLE	nd
*MdHsfB4a*	21-114	183-239	(325) KNTK-X_9_-KKR	(366) LEKDDLGLQLM	nd
*MdHsfB4b*	21-114	180-240	(327) KNTK-X_9_-KKR	(368) LEKDDLGLHLM	nd
*MdHsfC1a*	7-100	119-171	(195) KKRR	nd	nd
*MdHsfC1b*	9-102	128-180	( 204) KKRR	nd	nd

Number in brackets indicates the position of the first amino acid present in the putative nuclear localization signal (NLS), nuclear export signal (NES) and activator (AHA) motifs in the C-terminal domains. nd, no motifs detectable by sequence similarity searches.

**Figure 2:**
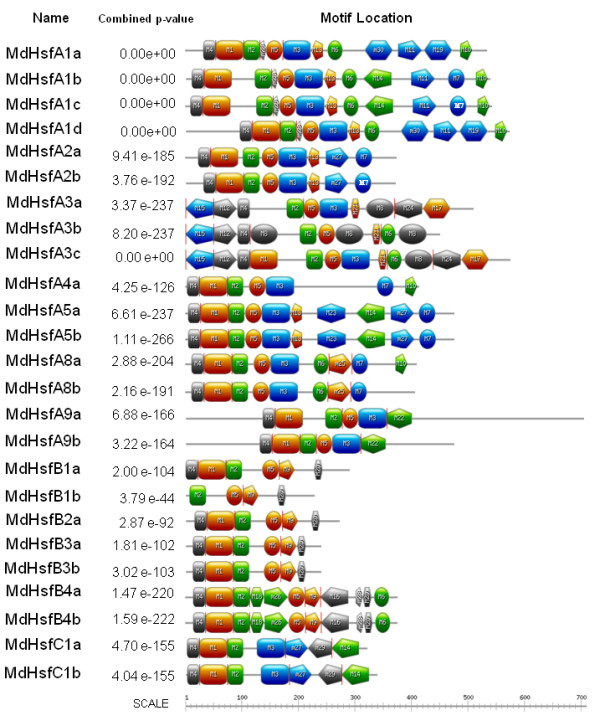
**Motifs identified by MEME tools in apple Hsfs.** Thirty motifs were identified and indicated by increasing number from 1 to 30. Their distribution and visualization on the full length protein sequences was performed by using the Expasy mydomain tools.

A second approach was used to identify and to verify domain prediction in the MdHsf proteins, by using the MEME motif search tool. Thirty corresponding consensus motifs were detected (Figure 2; Table 3). The majority of MdHsfs displayed the presence of the motifs 1, 2, 3, 4, 5 which correspond to highly conserved regions including the DBD and HR-A/B region domains. In addition, the inspection of motif distribution revealed that some of them were only present in specific classes of the MdHsf family. For example, motif 10 was representative of A-type Hsf members such as MdHsfA1a-A1d, MdHsfA4a, MdHsfA8a, and it contained the signature domains corresponding to NES sequence. Similarly, motif 7 containing the AHA sequence was detected in the C-terminal parts of many MdHsf proteins, belonging to the A class. Furthermore, eight A-type Hsfs members, namely MdHsfA1a-A1d, MdHsfA2a-b, MdHsfA5a-b were characterized by the presence of motif 13 which contained the NLS domains. Interestingly, all B Hsfs members exhibited motif 20, while MdHsfC1a and MdHsfC1b contained the motif 29 (Table 3).

**Table 3:** Motif sequences identified by MEME tools in apple Hsfs

**Motif**	**Length**	**Best possible match**
1	50	TDHIISWSDANNSFVVWDPPEFARDLLPKYFKHNNFSSFIRQLNTYGFRK
2	29	VDPDRWEFANEWFQRGQKHLLCNIHRRKH
3	50	LMQEIVRLRQQQQYTQNQLHAMNQRLQGMECRQQQMMSFLAKAMHNPGFL
4	21	LHKTGPPPFLCKTYDMVDDPA
5	29	YQQNPTGACVEVGKCGLWDEIERLKRDKN
6	26	CHKYMDGQIVKYQPPMNEAAKAMLRP
7	29	TPYTHPDIVNDIFWEQFWTARPICGNIEE
8	50	FEQSPHYPSQVTTGKLGLDAESTAFQFVDAALDELAITQGFLETPEQEGE
9	28	MLMSELAHMKKKCNEIIYFVANYVCMAW
10	20	GWDKSQNMNHITEQMGHLTS
11	41	QTDVVIPELTRIQGIVPEGNVDIPNANMIGEDIGNGFYMGM
12	41	EFEAFCSVNPLGAFDFTEKVSIPTSSMGGGGAEDVVVPPQP
13	20	HVHKNEKNRRITGYNKKRRL
14	49	IINPDAMLITKAPTGATNTRNSSQPGYGYTNGGGGHISCEVNYPTESTP
15	50	MSPKDESHPKSPPTSAEFDPESIGLSEFRPQVSAPLLGSQPIPSFTSPVM
16	50	PSNSYPSSMLLCNPQPPKHNGPNGNLNQLQGYYPAAPPPNAKQNPHHIMN
17	49	MIKQEDIWSMGFGVSAGMSTSMHELWGNPVNYDVPEMGVTGGLLDVWDI
18	26	AQPHQVGLNHHHHHHSPLGMNGHHHH
19	50	DGFIDPTSEVMNGSLPIDFDDISSDIEAFLKDWDDIIQNPGADEMDSTCA
20	14	EEECKNLKLFGVWL
21	15	VRRKFVKHQQHELSK
22	45	QQLMQKRMIKRELDGGDLGKRRRLPPAQGIESFDEWINDSLSFDC
23	50	FHQDFSSKLRLELSPAVSDMNLVSRSTQSSNEDGGSSTRKISEELKGAQM
24	50	GASSMVTEDPFFKGKSVLSPQQEANPERYVSFQEDLVKDRTFPELFSPGM
25	41	NSGSEKQPEVDAYMDGMEDFVVNPDFMKMLMDEKLSPVENH
26	41	FFPFPSRGSISPSDSDEQPNWCDSDSPPLLSPTGGINTNIN
27	40	PRMIQEIDYSAAAELGEKAKMVMMIAFTSSTAADDDKTTT
28	11	THVHDHQQQPP
29	41	ISSSPEAGFEMESFNRYPTPPEVQTASDWLRQRWFVDRVRA
30	50	CVSGVTLQEVPLTSGHGLPSVISETHSPPRVANPGTVMRSPFSDVNALVG

Numbers in the first column indicate the motifs represented in the Figure 2.

### Phylogenetic analysis of apple Hsf proteins

**Figure 3:**
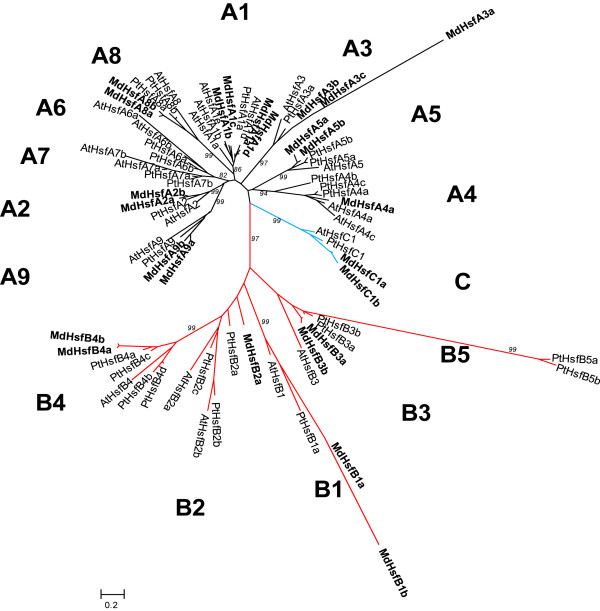
**Neighbor**-**joining phylogeny of Hsfs from *M. domestica, P. trichocarpa *and *A. thaliana.*** The phylogenetic tree was obtained using the MEGA 5.0 software on the basis of amino-acid sequences of the N-terminal domains of MdHsfs including the DNA-binding domain, the HR-A/B region and the linker between both regions. The final dataset included a total of 281 positions. Evolutionary distances were computed using the Jones-Taylor-Thornton matrix-based method and by removing all ambiguous positions for each sequence pair. Numbers indicate bootstrap values >80 based on 1000 replicates for the major nodes. The abbreviations of species names are as follows: Md, *Malus domestica*; Pt, *Populus trichocarpa*; At, *Arabidopsis thaliana.*

To investigate the evolution of Hsfs an unrooted phylogenetic tree was generated by using the 25 *Malus domestica* Hsfs, 28 *Populus trichocarpa* Hsfs (PtHsfs) and 21 *Arabidopsis thaliana* Hsfs (AtHsfs). *Populus* and *Arabidopsis* were chosen because their full sequence genome has been released, and Hsf members have been well characterized [7,10]. Moreover, the former is a tree. Figure 3 shows the result of this analysis. Hsfs of *Malus domestica*, *Arabidopsis thaliana* and *Populus trichocarpa* were clearly grouped into three different clades corresponding to the main Hsf classes A, B and C. Within the A-type clade, nine distinct sub-clades were resolved, seven of which (A1, A2, A3, A4, A5, A8 and A9) comprised the apple Hsf sequences. The C-type Hsfs from the three plant species also constituted one distinct clade which appeared more closely related to the Hsf A-group. Correspondingly, the B-type Hsfs from the three plant species grouped in a separate clade. Two of the five sub-clades, B3 and B2, were paraphyletic. As expected, the duplicated Hsfs of *Malus domestica* clustered all together on the phylogenetic tree.

### *In silico* expression analyses of *MdHsf* genes

Tissue specific expression of *MdHsfs* was investigated by counting the number of ESTs per tissue from EST libraries [17]. This resulted in the assignment of *MdHsfs* to nine groups on the basis of the tissue and organ types in which *MdHsfs* were present (Table 4).

**Table 4:** **Digital expression of *MdoHsf *genes**

**Tissue and organ type** (**DFCI Apple Gene Index**)									
**Gene name**	**Leaf**	**Root**	**Flower**	**Fruit**	**Shoot**	**Phloem**	**Xylem**	**Seed**	**Bud**
*MdHsfA1a*	+		+	+	+	+			
*MdHsfA1b*				+					
*MdHsfA1c*				+					
*MdHsfA1d*	+		+	+	+	+			
*MdHsfA2a*		+	+		+				
*MdHsfA2b*		+							
*MdHsfA3a*					+				
*MdHsfA3b*					+				
*MdHsfA3c*					+				
*MdHsfA4a*		+	+						
*MdHsfA5a*		+	+	+					
*MdHsfA5b*		+	+	+					
*MdHsfA8a*				+					
*MdHsfA8b*				+					
*MdHsfA9a*	+								
*MdHsfA9b*								+	
*MdHsfB1a*	+	+		+		+	+		
*MdHsfB1b*	+	+		+		+	+		
*MdHsfB2a*						+			
*MdHsfB3a*			+						
*MdHsfB3b*			+						
*MdHsfB4a*									+
*MdHsfB4b*									+
*MdHsfC1a*		+							
*MdHsfC1b*		+							

+: Expressed; blank: not expressed.

Of the group A1, *MdHsfA1a* and *MdHsfA1d*, were the most represented as their expression was detected in leaf, flower, fruit, shoot and phloem. Similarly, *MdHsfB1a* and *MdHsfB1b* of B class were expressed in several apple tissues. Interestingly, *MdHsfA9b* was the only Hsf specific for seed, whereas *MdHsfA9a* was found in leaf. Furthermore, expression restricted to only a single tissue type was observed also for other members of the *MdHsf* family; all A3-type *MdHsfs* were expressed in shoot and both members of the class C were found in root. In addition, the analysis of digital data showed that duplicated genes located on different chromosomes had identical expression patterns (*e.g. MdHsfB4a* and *MdHsfB4b*, *MdHsfC1a* and *MdHsfC1b*).

### Expression analysis of *MdHsf* genes in apple organs under natural environmental conditions

**Figure 4:**
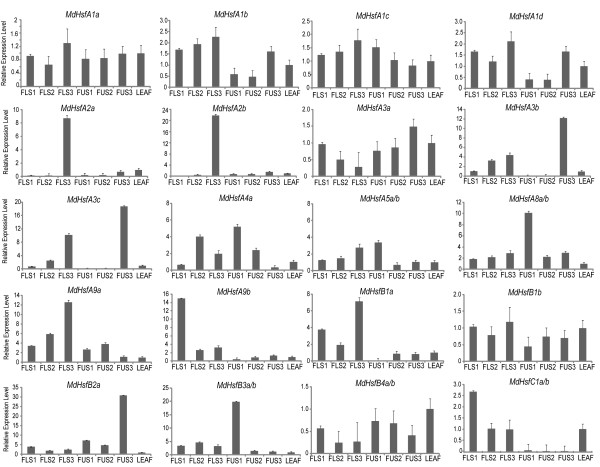
**Expression analyses of *MdHsfs *in developing flowers and fruits.** Quantification of messenger RNA levels was performed in developing flowers corresponding to the tight cluster, full pink and full bloom stages (FLS1, FLS2, FLS3) and in developing fruits of 10, 15 and 20 mm in diameter (FUS1, FUS2, FUS3). The relative expression of *MdHsf* genes in flower/fruit/different stages was calculated in relation to young leaves of 3–5 cm in length. The qRT-PCR analysis results were normalized using *EF1*, *Tip-41* and *IMPA9* as housekeeping genes. Each bar represents the average of the relative expression levels from three biological replicates.

*Hsf* genes are differentially expressed during flower and fruit development and are induced by abiotic environmental factors [7,12,13]. To investigate if *MdHsfs* are also involved in these processes, a comprehensive analysis of their expression was performed in flowers and fruits from field-grown trees. Flowers were harvested at the stages of tight cluster, full pink and anthesis (FLS1, FLS2, FLS3) during spring at average temperatures of 23°C/7°C (day/night; max/min), while the developing fruits were chosen at the stage of 10, 15 and 20 mm in diameter (FUS1, FUS2, FUS3) and harvest at average temperatures of 23°C/14°C (day/ night; max/min). Quantitative real-time PCR was used as the approach to monitor gene expression changes, and *MdHsf* transcript abundance in developing flowers/fruits was compared to that of vegetative leaf tissue (3–5 cm in length). The transcriptional patterns could be analyzed only for 20 *MdHsf* genes, since it was not possible to design specific primers discriminating *MdHsfA5a-b*, *MdHsfA8a-b*, *MdHsfB3a-b*, *MdHsfB4a-b* and *MdHsfC1a-b* because of high sequence similarity with the corresponding isoform. Figure 4 shows the results of this analysis. Members of A1 subgroups such as *MdHsfA1a-d* exhibited transcript accumulation in all tissues analyzed, although a higher induction was observed in flowers especially at anthesis. Similar to the A1-subgroup, higher messenger RNA levels at anthesis were also observed for other members of A class such as *MdHsfA2a-b*, *MdHsfA3b-c*, *MdHsfA5a-b* and *MdHsfA9a*. Interestingly, *MdHsfA9b* was approximately 4-fold more strongly induced in the youngest flowers than at the later stages. A broad variability of expression patterns in flowers and fruits was instead observed for the *MdHsf* members belonging to the B class. *MdHsfB1a* displayed a similar trend as a large part of the A members with higher expression at anthesis. On the contrary, its duplicated gene *MdHsfB1b* did not show any remarkable transcript changes between the different flower and fruit stages. *MdHsfB2a* showed strong up-regulation in 20 mm fruit where it was 30-fold higher than in leaf tissue. The B4-type gene *MdHsfB4a-b* exhibited a lower expression in flower/fruit than in leaf. Low transcript abundances in fruit as compared to flower or leaf were also observed for *MdHsfC1a-b*.

To further characterize the expression of Hsf family genes in apple, the quantitative real-time PCR analysis was extended to leaf samples harvested from field-grown trees exposed to naturally increased temperatures. Leaf samples were taken during the summer period, at two different temperature ranges: at 26°C/12°C (day/night; max/min) on 30th July 2011, which were used as reference, and at high temperature average of 32°C/17°C (day/night; max/min) on the 21st August 2011 (Additional file 1: Figure S1).

**Figure 5:**
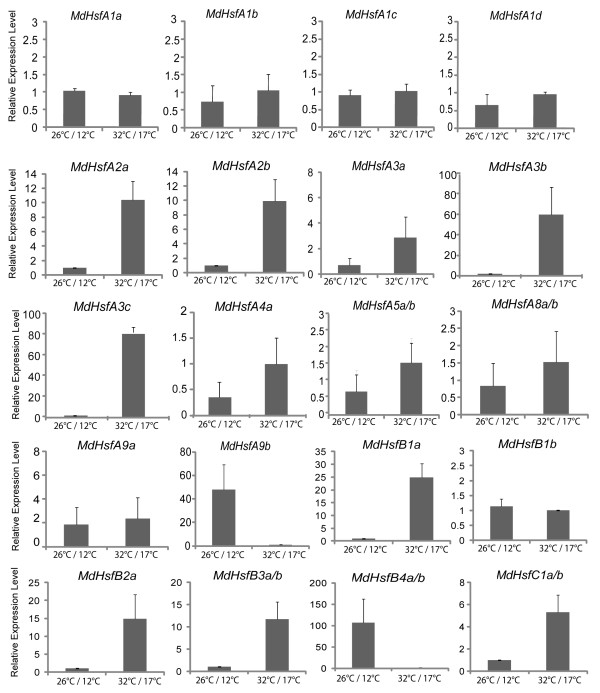
**Expression analyses of *MdHsfs *in vegetative leaf tissue under heat stress.** Messenger RNA levels of *MdHsfs* were analyzed by qRT-PCR in leaf samples from trees grown under field conditions and exposed to different temperature ranges: reference temperature, average of 26°C/12°C (day/night; max/min), considered as normal conditions; and high temperature, average of 32°C/17°C (day/night; max/min), considered as stress conditions. The qRT-PCR analysis results were normalized using *EF1*, *Tip-41* and *IMPA9* as housekeeping genes. Each bar represents the average of the relative expression levels from three biological replicates.

The transcriptional analyses revealed that in leaf most of the *MdHsfs* genes were responsive to the increased temperatures (Figure 5). Twelve of these responsive genes showed transcript accumulation significantly higher than the reference sample, while only *MdHsfA9b* and *MdHsfB4a-b* were strongly down-regulated in response to the increased temperatures. A 4-fold or higher increase of expression levels in response to high temperatures was observed for *MdHsfA2a-b*, *MdHsfA3b-c*, *MdHsfB1a*, *MdHsfB2a*, *MdHsfB3a-b* and *MdHsfC1a-b,* and only slightly higher in the stressed leaves than in the reference onces for *MdHsfA4a, MdHsfA5a-b* and *MdHsfA8a-b*. Furthermore, all subgroup A1 members such as *MdHsfA1a-d* did not show any significant transcriptional changes in response to the high temperatures compared to the reference conditions.

## Discussion

In plants, members of the Hsf family have been described as key regulators in molecular and cellular responses to stress conditions [1,7]. Furthermore, data from tomato and Arabidopsis have shown that the Hsfs are important components involved in developmental signalling [13,14]. Both size and composition of the Hsf family have been analyzed and characterized in different plant species [1]. The present study investigates for the first time this gene family in the economically relevant domesticated apple and shows that its genome contains 25 full length *Hsf* genes. This number is similar to that of *Populus trichocarpa* for which 28 loci encoding Hsf proteins were found [10]. Velasco et al. [2010] have shown that genome wide duplications had occurred in apple causing the expansion of several gene classes. Indeed, it was found that the enlargement of the *MdHsf* family is in particular originated from segmental duplications between different chromosomes. This situation is similar in maize and in *Populus,* in which segmental *Hsf* gene duplications were more prevalent than those of tandem duplications [9,10]. Gene duplications have an important role not only in the genomic rearrangement and expansion but also in diversification of gene function. In particular, genes encoding for nucleic acid binding proteins, among which transcription factors, originated mostly by segmental duplication. In contrast, membrane proteins and proteins involved in the stress response are encoded by genes mainly duplicated in tandem [18,19]. Therefore, the prevalence of segmental duplication events in *MdHsf* expansion may be associated to the fact that these genes act as transcriptional regulators.

*Malus*, *Arabidopsis* and *Populus* belong to the Rosid lineage and they are grouped in two distinct clades, namely Fabids (*Malus* and *Populus*) and Malvids (*Arabidopsis*) [20]. It was observed in the present study that the majority of the MdHsfs had a closer phylogenetic relationship to the PtHsfs than to the AtHsfs. This may be attributable to the fact that *Malus* and *Populus* belong to the same Fabids clade, and as they are both trees may have adapted to prolonged and repeated environmental constraints, unlike *Arabidopsis*.

Functional diversification of multifamily duplicated genes has been observed in trees. For example, the family of the glutathione S-transferase in *Populus* has a clear divergence in expression patterns in response to different stress treatments [21]. Therefore the presence of many duplicated *Hsf* genes in the apple genome may be related to the fact that a sub-functionalization has taken place especially to cope with prolonged and specific stress conditions.

*MdHsf* genes were found to be expressed in several apple tissues. In particular, members belonging to the A1 and B1 subclasses, such as *MdHsfA1a*, *MdHsfA1d*, *MdHsfB1a, MdHsfB1b*, were constitutively expressed in different tissues. A similar situation was found in other plants like *Arabidopsis* where A1-type Hsfs were involved in house-keeping processes under normal conditions, being ready for the fast activation of other Hsfs genes following stress treatment [22,23]. Furthermore, expression data from flower and fruit tissues indicated that some duplicated gene pairs, *e.g. MdHsfA9a* and *MdHsfA9b*, exhibited differences in their expression levels. This suggests that they may be subjected to a different regulation in apple tissue [1,7].

In contrast, the expression of *MdHsfA2a* and *MdHsfA2b* was mainly detected in full bloom flowers. AtHsfA9 and LeHsfA2a (Le, *Lycopersicon esculentum*) were found expressed in seed and developing pollen grains [13,14,24]. It was shown that the presence of these Hsfs during plant development is important for heat shock protein activation. This suggests that *MdHsfA2a* and *MdHsfA2b* may be important during pollination and fertilization, which occurs at anthesis.

Effects of heat stress (HS) on *Hsf* gene expression has been examined in several plant species, but no data are available about *Hsf* expression in trees exposed to naturally increased temperatures. Under laboratory settings, it was shown that AtHsfA1a and AtHsfA1b regulate the early response to HS in Arabidopsis [22,25]. AtHsfA2 is rapidly induced by HS, and it is involved in enhancing and maintaining of HS-response when plants are exposed to prolonged or repeated cycles of HS [26,27]. Similarly to AtHsfA2, AtHsfA3 is involved in thermo-tolerance mechanisms [7,28,29]. The A1-type *MdHsfs* were expressed at the same level also in leaves from plants growing in field and exposed to different temperature conditions. *MdHsfA2ab*, *MdHsfA3bc* were instead strongly induced. This may suggest that these types of *MdHsfs* could be involved in maintaining the stress response when apple trees are exposed to prolonged periods of high temperature conditions.

In contrast to class A Hsfs, genes assigned to the B and C classes have so far not been fully characterized. Members of the B class were shown to act mainly as repressors of the expression of HS inducible genes [30,31]. Some of them form a complex with Hsf A-types to maintain housekeeping gene expression during HS regimes [32]. Therefore, the strong transcriptional activation in apple may indicate that some of them may have a role in the response to the high temperatures also in this species. For the majority of *MdHsfs*, increased messenger RNA levels were observed under naturally increased temperatures. However, *MdHsfA9b* and *MdHsfB4a-b* were the only *Hsf* genes showing low transcript abundance. Although proteomic data are not available for all *MdHsfs* genes, their activation or repression may suggest that these transcripts could have a high hierarchy of molecular events induced by the high temperatures.

## Conclusions

The complexity of the Hsf family has been object of many investigations in different plant species. Here, 25 full length *Hsfs* genes were identified in the apple genome. Based on structural characteristics of the proteins and on the comparison with homologues from other species, the 25 MdHsfs were grouped in three different classes. Segmental and tandem duplications were examined and contributed to the expansion of the Hsf family in the apple genome. The expression profiles in flowers/fruits at different developmental stages as well as in leaves exposed to naturally increased temperature indicated that *MdHsfs* may play a role in different aspects of apple growth/development.

*Malus domestica* represents an economically important woody plant whose genome has been fully sequenced and whose commercial value is due to fruit production in the field. Therefore, understanding the role of protective genes as the *Hsfs* during development and under stress conditions is important. The results of this research will be undoubtedly useful for future gene cloning and functional studies and, in turn, for producing apple cultivars with improved genetic traits.

## Methods

### Identification and classification of Hsfs in *Malus domestica*

The recently sequenced apple genome was investigated for putative genes encoding for MdHsfs (Md: *Malus domestica)* based on BLASTN and BLASTP in NCBI and TIGR-Apple databases [11,15]. Physical localization of all candidate *MdHsfs* was analyzed in order to reject redundant sequences with the same chromosome location. In order to identify signature domains, the MdHsf sequences were compared to the Hsf proteins of Arabidopsis and tomato by amino acid sequence alignment using ClustalW (version 1.83). Presence of DBD domains and coiled-coil structures were checked by SMART and MARCOIL programs [33,34]. In addition, identification of putative domain motifs in the full-length amino acid sequences of the MdHsfs was also performed by MEME tools [35]. Visualization of the Meme motifs in the MdHsfs was performed by using Expasy tools (http://prosite.expasy.org/mydomains). MdHsf names were assigned on the basis of the original nomenclature as worked out for the *Arabidopsis thaliana* Hsf family, and later applied to other plant Hsf families [2,7]. Classification into three different groups A, B and C was based on the information of oligomerization domains [2].

### Phylogenetic analysis and gene duplication of *MdHsfs*

Gene duplications in the apple genome were analyzed by testing the similarity of all *MdHsf* genes using ClustalW. A gene duplication was defined according to the following criteria: (1) the length of the sequence alignment covered ≥ 80 % of the longest gene, and (2) the similarity of the aligned gene regions was ≥ 80 % [36,37]. Data were then plotted using Circos software [38].

To understand the evolutionary relationships of the MdHsf proteins, a phylogenetic tree was constructed. The N-terminal Hsf protein sequences containing the DBD and HR-A/B regions from *Malus domestica*, *Arabidopsis thaliana and Populus trichocarpa*[7,10] were aligned using ClustalW. A phylogenetic tree was constructed using the Neighbor Joining (NJ) method in MEGA (version 5.0) [39]. Based on the results of the model selection analysis, the Jones-Taylor-Thornton matrix-based method was used to compute evolutionary distances [40]. The rate variation among sites was modeled with a gamma distribution (shape parameter = 0.67). Bootstrap analysis was conducted with 1000 replicates to assess statistical support for each node.

### Digital and EST expression analysis

The analysis of *MdHsfs* expression profiles was investigated at the transcriptional level. *MdHsfs* expression patterns were searched with the BLAST program in TIGR-Apple EST libraries [17] using the following parameters: maximum identity > 95%, length > 200 bp and E-value <10^-10^.

### Plant material

Experiments were carried out in 2011 on 18-year-old apple trees (cultivar ‘Golden Delicious’ on M9 rootstock) trained with standard horticultural practices at the experimental farm of the Research Centre for Agriculture and Forestry Laimburg (South Tyrol, Italy). Samples were taken from 24 homogeneous trees grouped in 3 biological replicates each containing 8 trees distributed in the same block of the orchard. Tissue samples were collected between April and August 2011 from trees grown under field environmental conditions and exposed to natural variations of temperature and solar radiation. Temperature data are reported in the Additional file 1. Young leaves (3–5 cm in length) as well as developing flowers corresponding to the tight cluster (FLS1), pink (FLS2) and full bloom (anthesis, FLS3) stages were harvested from the plants during spring period and under max-minimum temperature average in the range of 23°C/7°C (day/night; max/ min). From the same trees developing fruits of 10 mm (FUS1), 15 mm (FUS2) and 20 mm (FUS3) in length were also collected under max-minimum temperatures of 23°C/14°C (day/night; max/min). For testing *Hsfs* gene expression under naturally increased temperature conditions, leaf samples were taken during the summer period, at two different temperature ranges: at 26°C/12°C (day/night; max/min) on 30th July, 2011, which were used as reference, and at high temperature average of 32°C/17°C (day/night; max/min) on the 21st August, 2011 (Additional file 1: Figure S1). All samples used in gene expression analyses were harvested at midday (12:00 am) and were positioned around 1.60 m in height from the soil.

### RNA isolation and quantitative real-time PCR (qRT-PCR) analyses

Total RNA was isolated from apple tissues with the hot phenol method [41]. RNA quantity was measured using a NanoDrop ND-1000 spectrophotometer, and its quality was checked by agarose gel electrophoresis. For reverse transcription, total RNA was incubated with RNase-free DNase (RQ1; Promega, Madison, WI), and 1 μg was used for reverse transcription according to the manufacturer’s instructions (Superscript Vilo cDNA Synthesis kit; Invitrogen).

The qRT-PCR analyses were carried out on a 7500 Fast Real-time PCR System (Applied Biosystems) with the ROX Reference Dye. Each reaction contained 12.5 μl SYBR GreenER qPCR SuperMix Universal (Invitrogen), 20 ng of cDNA and 400 nM of each specific primer. The qRT-PCRs were performed using a controlled temperature program starting with 10 min at 95°C, followed by 40 cycles of 15 s at 95°C and 60 s at 60°C. To verify the presence of a specific product, the melting temperature of the amplified products was determined. In addition, each PCR mixture was analyzed on a 2% agarose/ethidium bromide stained gel to verify the size of the amplified DNA fragment. The primers used for the qRT-PCRs were designed using Quantprime software and are reported in the Additional file 2[42]. The qRT-PCRs were performed in duplicated technical reactions and repeated on three independent biological replicates. Relative mRNA levels of the target genes were calculated based on Vandesompele et al. [2002] [43]. The genes encoding for elongation factor 1 alpha subunit (eF-1 alpha; accession number *AJ223969.1*), Importin alpha Isoform9 (IMPA-9; accession number *CN909679*) and Tip-41 like protein (Tip-41 *CN941833*) were used as references in the qRT-PCR analyses.

## Abbreviations

Hsf: Heat shock transcriptional factor; HR-A/B: Adjacent bipartite oligomerization domain; AHA: Activator motif; CTAD: C-terminal activation domain; DBD: DNA-binding domain; HS: Heat stress; HSE: Heat shock element; NES: Nuclear export signal; NLS: Nuclear localization signal; qRT-PCR: Quantitative reverse transcription real-time PCR.

## Competing interests

The authors declare that they have no competing interests.

## Authors’ contributions

This study was designed by FG, SB and CM. Bioinformatics analyses were performed by FG, expression analyses by FG and GG. FG and GG assisted to tissue sampling. FG, GG, SB and CM contribute to manuscript preparation and revision. All authors have read and approved the final manuscript.

## Supplementary Material

Additional file 1: Figure S1 Average temperature in the orchard where apple trees were sampled during the 2011 growing season. The data show temperature ranges obtained from a meteorological station located in the apple orchard and positioned around 2 m in height. Each point represents the average calculated on the basis of data from seven days. Click here for file

Additional file 2 Primer sequences used for quantitative real-time PCR analyses. Click here for file
