# Supplementary material for: Heat shock transcriptional factors in Malus domestica: identification, classification and expression analysis
Source: BMC Genomics. 2012 Nov 20;13:639. doi: 10.1186/1471-2164-13-639 (PMC3575323; doi:10.1186/1471-2164-13-639)

Additional file 1-Temperature average in the orchard where apple trees were grown during 2011 season.

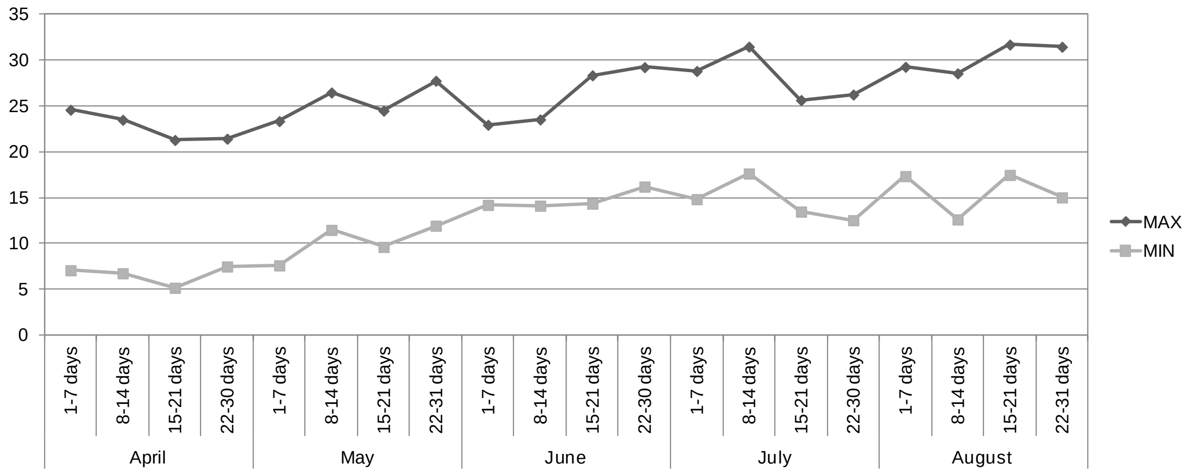

Supplement: Additional file 1: Figure S1 — Average temperature in the orchard where apple trees were sampled during the 2011 growing season. The data show temperature ranges obtained from a meteorological station located in the apple orchard and positioned around 2 m in height. Each point represents the average calculated on the basis of data from seven days. [file 1471-2164-13-639-S1.pdf]
