# Supplementary material for: Heat shock transcriptional factors in Malus domestica: identification, classification and expression analysis
Source: BMC Genomics. 2012 Nov 20;13:639. doi: 10.1186/1471-2164-13-639 (PMC3575323; doi:10.1186/1471-2164-13-639)
Supplement: Additional file 2 — Primer sequences used for quantitative real-time PCR analyses. [file 1471-2164-13-639-S2.pdf]

Additional file 2 – Primer sequences used for quantitative real-time PCR analyses.

Gene Name	Primer sequence
MdHsfA1a Forw	GCATCTGAAGCAGGTGATGG
MdHsfA1a Rev	GCTGCTGCTCTTCATTTTCC
MdHsfA1b Forw	TCGAATGCAAGGTATTGTTCC
MdHsfA1b Rev	TGCTTGGAAGGCTAGAGACC
MdHsfA1c Forw	ATGTCAGCAGGTTTGGATGG
MdHsfA1c Rev	TGCAAATCCTGATTCACAGC
MdHsfA1d Forw	GTCATCCGAAGGCTTAGTGG
MdHsfA1d Rev	CATCCCAATCCTTTAGAAATGC
MdHsfA9a Forw	AGGTGGCTCCGTCCAAATA
MdHsfA9a Rev	TCGAGACGAAGACACTCCAA
MdHsfA2a Forw	TGCTGCATTGGACAACAAAT
MdHsfA2a Rev	GCAACCAAATCATCCACCTC
MdHsfA2b Forw	CTGCATTGGACAACAAATCG
MdHsfA2b Rev	AACCAAATCCTCCACCTCAA
MdHsfA9b Forw	ATTGTGGAGGCCAAATTGAG
MdHsfA9b Rev	CATCATAAGCAGAGGGCACA
MdHsfA3a Forw	TTTCGGTGTCAGTGCTGGTA
MdHsfA3a Rev	ACTCTCGGGCTCATCAAATG
MdHsfA3b Forw	CTGCTTGGAGAAACCAAACC
MdHsfA3b Rev	TTCGCCTTCTTGCTCTGG
MdHsfA3c Forw	TGATGTCAGTGCCGGTATGT
MdHsfA3c Rev	ATGCACACTGGACACCTTGA
MdHsfA4a Forw	CAATGACGGATTTTGGGAAC
MdHsfA4a Rev	GAGTGAGATGCCCCATCTGT
MdHsfA5a/b Forw	ACAATCAAGCACCTGCACTG
MdHsfA5a/b Rev	GTGAGTGTTTCCGCATCCTT
MdHsfA8a/b Forw	GCCTGCTGATGCTAGAATGG
MdHsfA8a/b Rev	GCTTCCCTGATTCTGTTCCA
MdHsfB1a Forw	GCGATGATGACGAAGAAGAA
MdHsfB1a Rev	ACACACCTTCCCACTCCTCA
MdHsfB1b Forw	GGAAAGGCTTGGAGGACATT
MdHsfB1b Rev	CAGCATCAGCCCCAAATAAT
MdHsfB2a Forw	AGCATGGTCTCGAATTACGC
MdHsfB2a Rev	CGGTCTCATCCTCCTCTACG
MdHsfB3a/b Forw	AACAAAAGGCTGAAGCAGGA
MdHsfB3a/b Rev	TGCGGTTGCACTAATTTCAG
MdHsfB4a/b Forw	TCCTCCACCTAATGCCAAAC
MdHsfB4a/b Rev	GTACTCCTCCGGGTGCAAT
ModHsfC1a/b Forw	TCGGGTTCACGCTATACAAA
ModHsfC1a/b Rev	TCCCTCCAATAGCGAAAATG

eF-1alpha Forw	ACTGTTCCTGTTGGACGTGTTG
eF-1alpha Rev	TGGAGTTGGAAGCAACGTACCC
IMPA-9 Forw	TCGTGAACTCAGGCGCTTACTG
IMPA-9 Rev	AAGCAACGGTAAAGCGGGCAAC
TIP41 Forw	ACATGCCGGAGATGGTGT TTGG
TIP41 Rev	ACTTCCAGAGTACGGCGTTGTG
